# Laparoendoscopic single-site varicocelectomy compared with conventional laparoscopic surgery: a systematic review and meta-analysis

**DOI:** 10.1186/s40064-016-3178-1

**Published:** 2016-09-05

**Authors:** Tao Wu, Xi Duan, Xuesong Yang, Xianzhong Deng, Shu Cui

**Affiliations:** 1 Department of Urology, Affiliated Hospital of North Sichuan Medical College, Nanchong, 637000 China; 2 Department of Dermatovenereology, Affiliated Hospital of North Sichuan Medical College, Nanchong, 637000 China

**Keywords:** Varicocelectomy, Laparoscopic, Laparoendoscopic single-site surgery, Meta-analysis

## Abstract

**Purpose:**

To present a systematic review and meta-analysis comparing laparoendoscopics single-site varicocelectomy (LESS-V) versus conventional laparoscopic surgery (CTL-V).

**Methods:**

A literature search was performed using The Cochrane Library, MEDLINE, EMBASE, Science Citation Index Expanded and Google Scholar. Literature reviewed included meta-analyses, and randomized and nonrandomized prospective studies. We utilized weight mean difference (WMD) to measure hospital stay, time to return normal activity, postoperative pain and improvement of semen parameters and odds ratio (OR) to postoperative complications and cosmetic satisfaction. We used the Cochrane Collaboration’s Review Manager 5.1 software for statistical analysis.

**Results:**

We identified six publications which strictly met our eligibility criteria. Meta-analysis of extractable data showed that LESS-V was better than CTL-V in postoperative pain (WMD: −0.46; 95 % CI −0.75 to −0.17; p = 0.002), time to convalescence (WMD: −1.4 days; 95 % CI −2.55 to −0.25; p = 0.02) and cosmetic satisfaction (OR 6.86; 95 % CI 2.89–16.28; p < 0.00001). However, CTL-V was better than LESS-V in operative time (WMD 1.96 min, 95 % CI 0.96–2.96; p = 0.0001). There were no differences between LESS-V and CTL-V in hospital stay (WMD: −0.02 days, 95 % CI −0.39 to 0.35; p = 0.92) and postoperative complications (OR 1.13, 95 % CI 0.57–2.21; p = 0.73).

**Conclusions:**

This meta-analysis comparing the efficacy of LESS-V and CTL-V showed that LESS-V was safe, with significantly reduced postoperative pain, shorter recovery time, and better cosmetic outcome.

## Background

Varicocele is defined as dilatation of pampiniform plexus of scrotal veins. It is present in 13 % of the normal male population and in approximately 37 % of men with infertility (Clarke 1966). Surgical repair of varicocele can improve sperm parameters, testosterone production, and fertility (Daitch et al. 2001; Baazeem et al. 2011; Schlegel 1997). There are several surgical techniques to treat varicoceles, including open inguinal, subinguinal microscopic, and laparoscopic ligation (Ding et al. 2012; Al-Kandari et al. 2007). However, the ideal approach of varicocele treatment is still a matter of controversy (Cayan et al. 2009). Among the various approaches of repair, microsurgical surgery seems to be associated with better outcomes (higher spontaneous pregnancy rates and lower postoperative recurrence) (Baazeem et al. 2011; Cayan et al. 2009). But the operating times for microsurgical repair are significantly longer than for laparoscopic procedures. There is no difference between the microsurgical and laparoscopic techniques in complication rates in long-term (VanderBrink et al. 2007; McManus et al. 2004). Further, microsurgical repair might require microsurgical training (Baazeem et al. 2011). Recent studies have shown that laparoscopic varicocelectomy is safe, effective, cost effective, minimally invasion and with a low recurrence rate (Borruto et al. 2010).

As one type of the laparoscopic surgery, the laparoendoscopic single-site (LESS) surgery has been developed in an attempt to further reduce the morbidity and scarring associated with surgical intervention (Fan et al. 2012; Autorino et al. 2011). Kaouk and Palmer reported the first umbilical LESS varicocelectomy (LESS-V) in 2007. Since then, more and more case reports and control studies comparing LESS with conventional laparoscopy have increased. Even though several studies comparing LESS-V and conventional laparoscopic varicocelectomy (CTL-V) have been reported, most are small series with conflicting results (Bansal et al. 2014; Friedersdorff et al. 2013; Lee et al. 2012; Marte et al. 2014; Wang et al. 2014; Youssef and Abdalla 2015). It is still uncertain whether the benefits of LESS-V are restricted to improved fertility and are superior to CTL-V. Our goal is to therefore systemically search and analyze the available literature to conduct a meta-analysis of these studies to compare LESS-V–CTL-V.

## Methods

### Study search strategy

We searched online databases, including The Pubmed, EMBASE, Science Citation Index Expanded, Cochrane Library and Google scholar to identify suitable studies until the end of October 2015, with no lower date limit. All initially identified studies were further filtered on the basis of predetermined relevant Medical Subject Heading (MeSH) terms and/or key words. The following MeSH terms and keywords were used: laparoscopy, varicocelectomy, varicocele, single site, single incision, varicocele ligation, varicocele repair, laparoendoscopic, LESS, single-site, single port, single access and human with every possible combination considered. We have tried to contact all corresponding authors when data were missing.

### Identification of articles and data abstraction

After the studies were reviewed, it was noted that none of the previously performed strict and organized meta-analyses and systematic that reported comparing LESS with CTL for varicocele. Some reviews compared open inguinal, subinguinal microscopic and laparoscopic ligation for the varicocele but it did not compare LESS-V–CTL-V. Those studies did not include the recent RCTs and not performed the correct meta-analyses either.

**Fig. 1:**
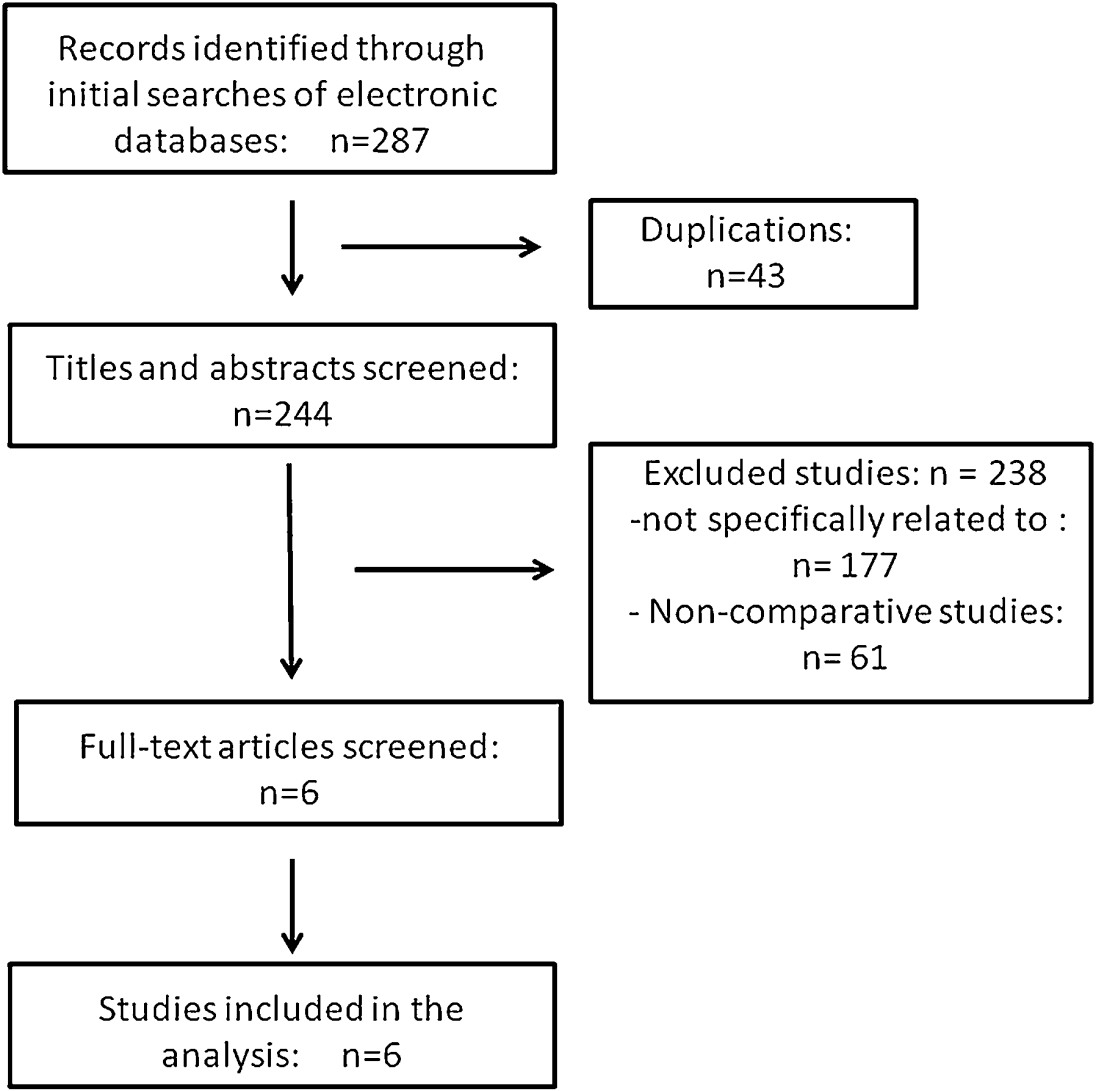
Flowchart for records selection process of the meta-analysis

The original published articles of 287 relevant citations were retrieved for full review by using inclusion criteria that entailed selection of any study (observational or clinical trials) of comparing LESS-V with CTL-V. We got six studies by this process (Fig. 1). Case reports that reported exclusively on LESS-V were excluded because of the large clinical diversity. No attempt was made to restrict the search according to more specific methodological characteristics. The studies were reviewed by two independent investigators (X.D. and X.Y.) to determine whether they met the eligible criteria for inclusion. Discrepancies for inclusion between the investigators were resolved by discussion. Only studies that met the following criteria were included: (1) compared LESS-V–CTL-V with quantitative data on outcome parameters; (2) had the similar inclusion baseline. Using a standardized form, we recorded procedural characteristics of each study, including type of design, level of evidence, number of participants and multichannel port for LESS.

### Quality assessment of included studies

Each included article was appraised by two reviewers (X.D. and X.Y.), who assessed the methodological quality of selected studies independently with the quality of included studies. RCTs were assessed by the Cochrane risk-of-bias tool, attributing ONE point to each item (total score range 0–8) (Higgins and Green 2008). Non-RCTs were assessed by Newcastle–Ottawa Scale (NOS)(Wells et al. 2000). Two reviewers assessed and scored the representative and applicability of study groups, comparability of the groups, evaluation of outcomes, and adequacy of follow-up. And we defined score of 6–9 was high methodological quality and low quality as a score <6.

### Data synthesis and data analysis

Meta-analyses were performed for primary and secondary outcome parameters: hospital stay, operating time, pain score, time to return to normal activity, postoperative complications. The Review Manager 5.1 software (The Cochrane Collaboration, Oxford, UK) statistical package was used to analyze the ORs for dichotomous variables and weighted mean differences (WMDs) for continuous variables. Depending on whether homogeneity was accepted or rejected, we used the fixed or the random effect model. Statistical heterogeneity was assessed by the Chi square test and was expressed by the I^2^ index as described by Higgins and colleagues. p values of <0.05 was considered to indicate statistical significance. I^2^ < 50 % indicated acceptable heterogeneity. The confidence interval (CI) was established at 95 %.

## Results

### Study characteristics and quality assessment

Table 1 depicts the study characteristics and methodology for the six studies included in the systematic review (Bansal et al. 2014; Friedersdorff et al. 2013; Lee et al. 2012; Marte et al. 2014; Wang et al. 2014; Youssef and Abdalla 2015). Among these, three were randomized controlled trials (level of evidence 2b) (Lee et al. 2012; Wang et al. 2014; Youssef and Abdalla 2015). Another three were retrospective case control studies (level of evidence 3b–4) (Bansal et al. 2014; Friedersdorff et al. 2013; Marte et al. 2014). All publications reported on similar outcomes (i.e., hospital stay, operating time, pain score, time to return to normal activity, complications). In all cases of missing or incomplete information, we contacted all the authors of the studies but none could provide any additional data.

**Table 1:** Characteristics of included studies

References	Design	Number of patients		Level of evidence	Multichannel port for LESS	Quality score (failing items)	NOS score (max: 9)
		LESS-V	CTL-V				
Bansal et al. (2014)	Retrospective case control study	11	32	3b	TriPort	–	5
Friedersdorff et al. (2013)	Retrospective case control study	20	79	3b	X-cone	–	6
Lee et al. (2012)	RCT	39	43	2b	Home-made	6 (BC)	–
Marte et al. (2014)	Retrospective case control study	44	25	4	SILS	–	5
Wang et al. (2014)	RCT	44	43	2b	Home-made	6 (BC)	–
Youssef and Abdalla (2015)	RCT	41	39	2b	SILS	6 (BC)	–

Quality items of RCTs according to Cochrane risk-of-bias tool (score range 0–8): A—adequate method of sequence generation, B—blinding of participants performed, C—blinding of personnel performed, D—blinding of assessors performed, E—allocation concealment adequate, F—adequate assessment of each outcome, G—selective outcome reporting avoided, H—intention-to-treat analysis of results

### Perioperative outcomes

#### Operative time and postoperative pain

**Fig. 2:**
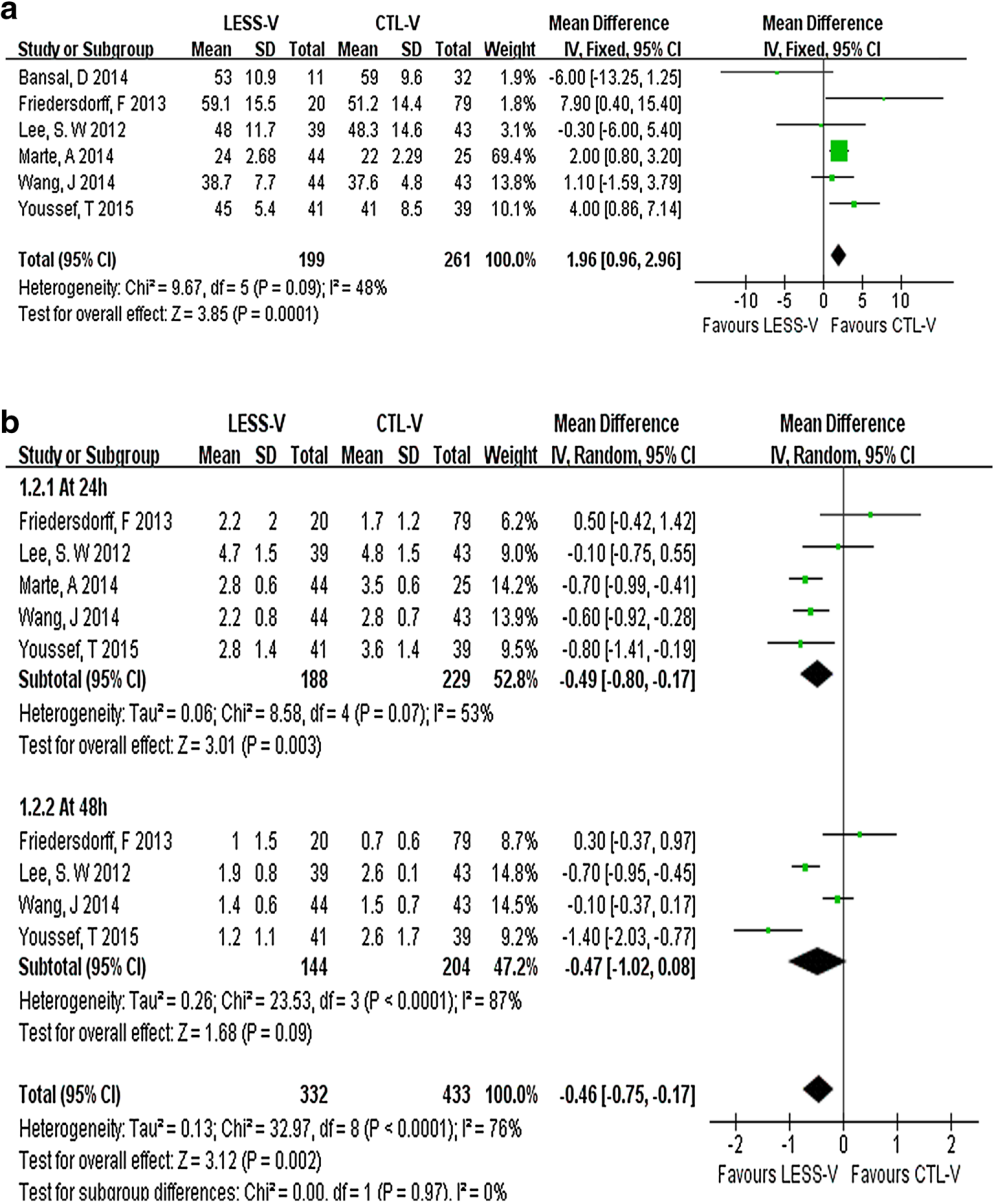
Pooled estimate of operative time (**a**) and postoperative pain (**b**) of LESS-V versus CTL-V. Each subgroup analysis is presented separately

All of six studies evaluated operative time. These trials involved 460 couples (199 for LESS-V and 261 for CTL-V). There was a significant shorter operative time in the CTL-V group (WMD: 1.96 min, 95 % CI 0.96–2.96; p = 0.0001; Fig. 2a). Five studies including 417 patients evaluated postoperative pain using the VAS at different time points, ranging from the first postoperative day to the day of discharge (Friedersdorff et al. 2013; Lee et al. 2012; Marte et al. 2014; Wang et al. 2014; Youssef and Abdalla 2015). Because of the difference of evaluating time points, the subgroup analysis was performed. The pooled data showed significant lower VAS scores in the LESS-V group than the CTL-V group (WMD: −0.46; 95 % CI −0.75 to −0.17; p = 0.002; Fig. 2b).

#### Hospital stay and time to return to normal activity

**Fig. 3:**
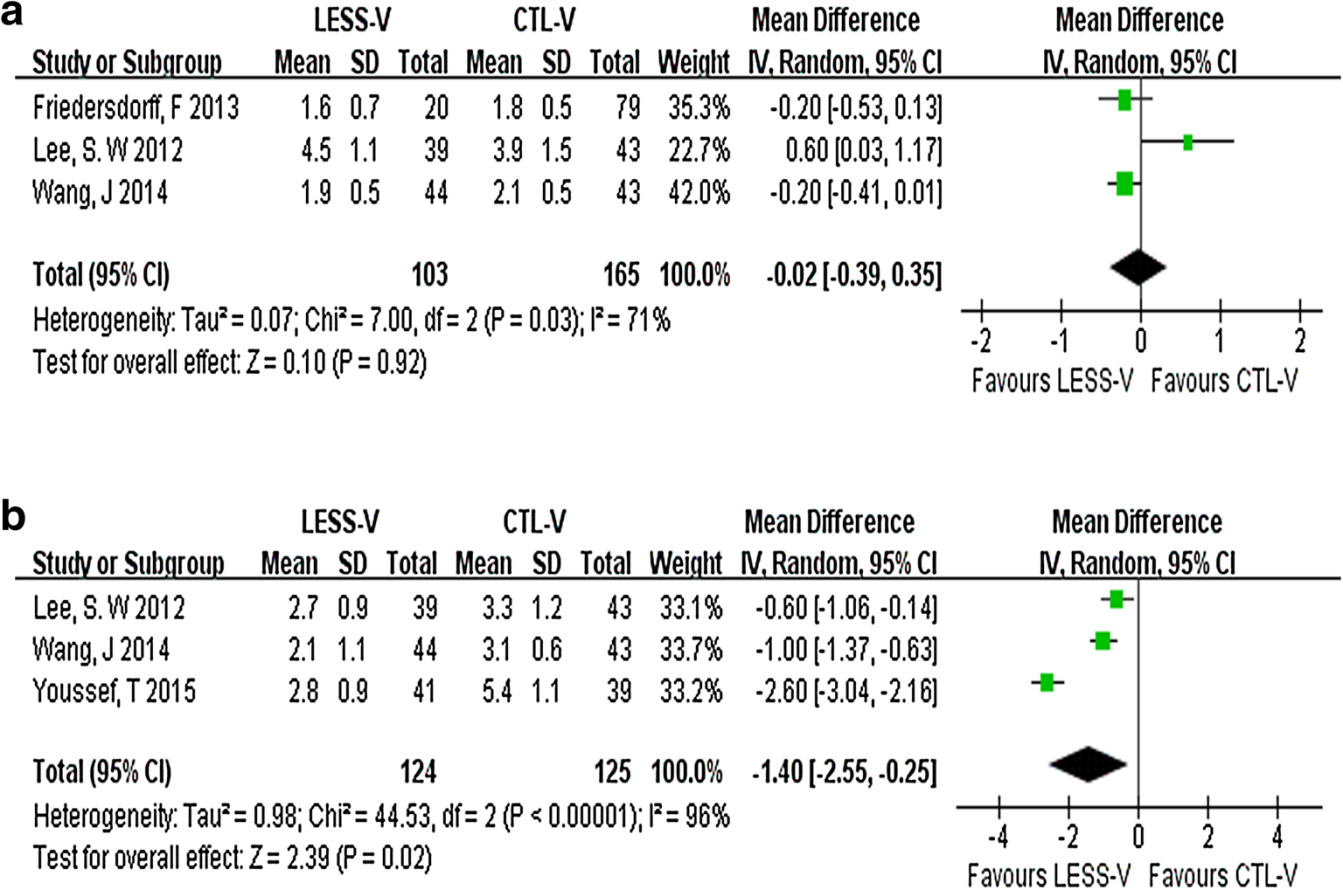
Pooled estimate of hospital stay (**a**) and time to return to normal activity (**b**) of LESS-V versus CTL-V

We identified three trials reporting hospital stay (Friedersdorff et al. 2013; Lee et al. 2012; Wang et al. 2014). These trials involved 268 couples (103 for LESS-V and 265 for CTL-V). The Random-effect model WMD was −0.02 (95 % CI −0.39 to 0.35; p = 0.92; Fig. 3a), suggesting that there is no significantly difference between LESS-V and CTL-V for hospital stay. Three trials reported time to convalescence in 249 patients (124 for LESS-V and 125 for CTL-V) (Lee et al. 2012; Wang et al. 2014; Youssef and Abdalla 2015). The pooled data showed a significant difference favoring the LESS-V group (WMD: −1.4; 95 % CI −2.55 to −0.25; p = 0.02; Fig. 3b).

#### Postoperative complications, improvement of semen parameters and cosmetic satisfaction

**Fig. 4:**
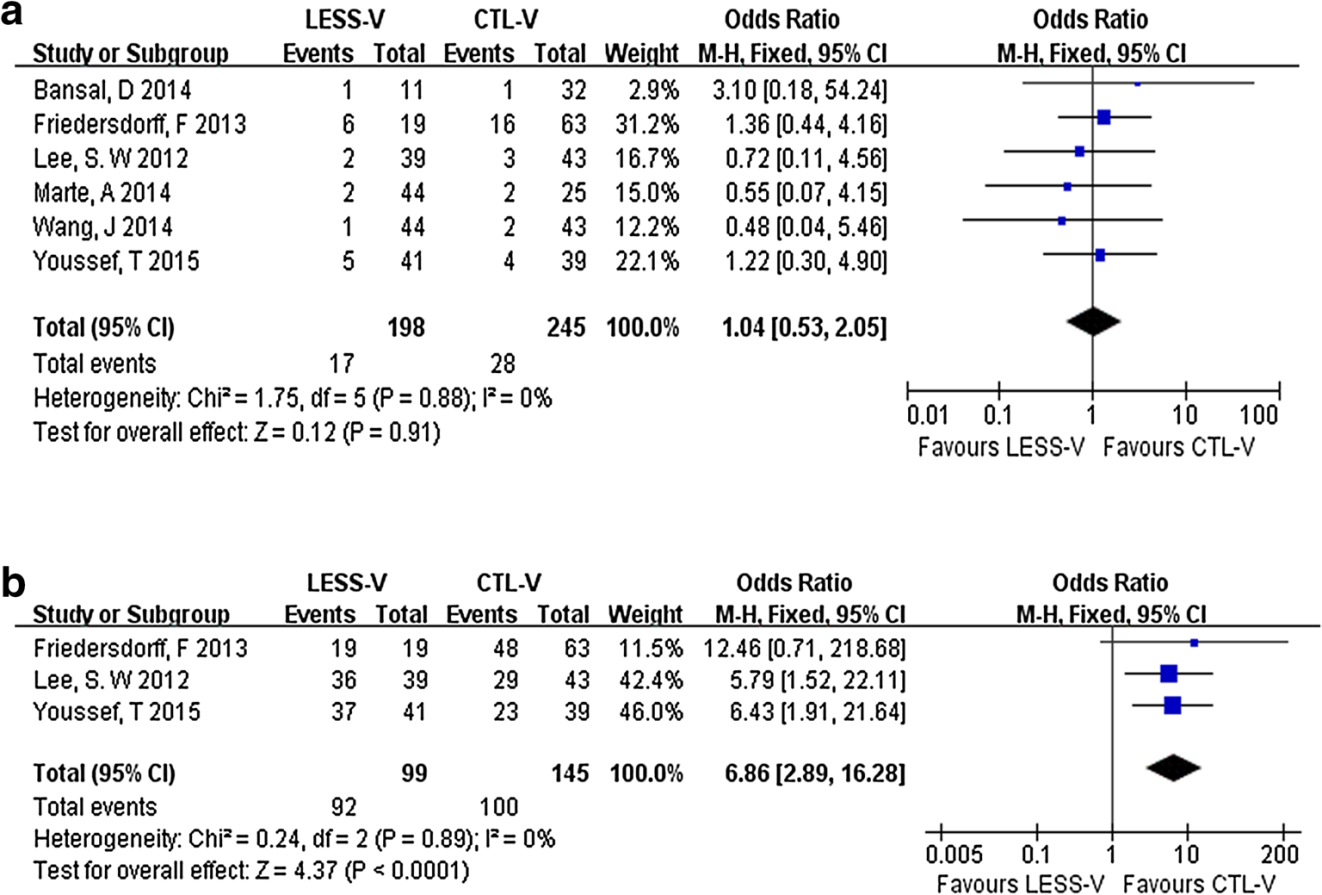
Pooled estimate of postoperative complications (**a**) and parameters and cosmetic satisfaction (**b**) of LESS-V versus CTL-V

Pooling the data from six studies that assessed postoperative complications in 460 patients (199 for LESS-V and 261 for CTL-V) showed no significant difference between the LESS-V and CTL-V groups. The fixed-effect model combined OR was 1.04 (95 % CI 0.57–2.21; p = 0.73; Fig. 4a). The most common complications are hydrocele and recurrent varicocele. Four studies reported cosmetic results (Friedersdorff et al. 2013; Lee et al. 2012; Wang et al. 2014; Youssef and Abdalla 2015). Three of them showed number of patients who satisfied with cosmetic outcome (Friedersdorff et al. 2013; Lee et al. 2012; Youssef and Abdalla 2015). Pooling the data of the 244 patients in these three studies showed significantly better cosmetic satisfaction in the LESS-V group than the CTL-V group (OR 6.86; 95 % CI 2.89–16.28; p < 0.00001; Fig. 4b). Another study reported verbal response scale and numeric scale to depict cosmetic results. These two scales all showed significantly greater satisfaction in the LESS-V group than the CTL-V group (verbal response scale: p = 0.008, numeric scale: p = 0.005) (Wang et al. 2014). Four trials evaluated semen parameters including: sperm count, motility and normal morphology (Friedersdorff et al. 2013; Lee et al. 2012; Wang et al. 2014; Youssef and Abdalla 2015). There is no significant difference in improvement of sperm parameters between LESS-V and CTL-V groups in each study. However, in these studies, both LESS-V and CTL-V groups could improve sperm parameters.

### Heterogeneity analysis

Our pooled estimates for these studies still had significant heterogeneity in comparison of hospital stay, time to return normal activity and postoperative pain score. Even after performing those subgroup analyses, the I^2^ test nonetheless remained to be >95 %, p < 0.001, attesting to heterogeneity that could not be explained, as shown in Figs. 1b, 2a, b. We attempted to explain heterogeneity by performing subgroup analyses involving different measuring time point of pain score; however it still had significant heterogeneity. Pooled data using the random-effect model might reduce the effect of heterogeneity but does not abolish it. Publication bias was reduced to the minimum according to our search strategy. We included all data strictly into our review and the baseline was maintained consistency. Data extraction forms from the six selected studies are displayed in Table 1.

## Discussion

To our knowledge, our meta-analysis of three RCTs and three retrospective studies including 460 patients comparing the efficacy of LESS-V and CTL-V showed that LESS-V was safe, with significantly reduced postoperative pain, shorter recovery time, and better cosmetic outcome. We found no significant differences in postoperative complications, hospital stay and improvement of sperm parameters.

Varicocele has an adverse effect on the histologic, endocrine, and testis function (Romeo and Santoro 2009). Varicocelectomy is indicated in the case of infertility, when the testicular volume is decreased, such as in adolescents, and when associated with persistent pain (Spinelli et al. 2010). There are several surgical procedures for varicocele. Two meta-analyses considered that the microsurgical varicocelectomy technique has higher spontaneous pregnancy rates and lower postoperative recurrence and hydrocele formation than conventional varicocelectomy techniques in infertile man (Ding et al. 2012; Cayan et al. 2009). LESS has been developed with the hypothesis that minimizing the number of skin incisions needed to gain access to the abdominal or pelvic cavities may benefit patients in regard to pain control, convalescence, cosmesis, and access related complications. Driven by these advantages, the world experience in urologic LESS is steadily increasing (Wang et al. 2013; Kaouk et al. 2011).

In the application of any new technique, the safety of the patients is always of most importance. The pooled data of postoperative complications indicates that the LESS approach is safe and effective for varicocele. There was no significant difference in postoperative complications between LESS-V and CTL-V group. The most common complications were hydrocele formation and varicocele recurrence. This was similar same as literature reported (Diegidio et al. 2011). Diegidio et al. (2011) reviewed current varicocelectomy techniques and their complication rate. They found hydrocele formation rates were lowest with microsurgical inguinal technique. Other studies compared open non-microsurgical, laparoscopic or open microsurgical varicocelectomy. They thought the incidences of recurrent varicocele and postoperative hydrocele was significantly lower after microsurgery than after laparoscopic or open varicocelectomy (Ding et al. 2012; Cayan et al. 2009). But the laparoscopic varicocelectomy they mentioned were all conventional laparoscopic methods. They did not compare LESS with other surgical techniques.

Operating time is routinely considered as a parameter to estimate the surgical learning curve. The pooled analysis of operative time showed there was a significant shorter operative time in the CTL-V group. The possible reason for that the features specific to the LESS technique (such as crossing or collision of instruments, lack of triangulation, and in-line vision) represent additional challenges for surgeons compared with conventional laparoscopy (Kaouk et al. 2011).

The rationale behind the adoption of LESS is mainly based on the potential gain for the patient in terms of lower postoperative pain, shorter hospital stay, and ultimately faster recovery (Autorino et al. 2015). The pooled data suggested that there is no significantly difference between LESS-V and CTL-V for hospital stay. But regarding time to convalescence, it showed a significant difference favoring the LESS-V group. One direct advantage of LESS-V concerns postoperative pain. In our meta-analysis, the postoperative VAS score in the LESS-V group was significantly lower than that in CTL-V group. As LESS-V reduces the number of skin incisions to only one, it seems reasonable to postulate that LESS is less invasive than conventional laparoscopy (Tracy et al. 2008).

Baazeem et al. (2011) considered although there is no conclusive evidence that a varicocele repair improves spontaneous pregnancy rates, sperm parameters (count and total and progressive motility), reduces sperm DNA damage and seminal oxidative stress, and improves sperm ultra-morphology. The various methods of repair are all viable options, but microsurgical repair seems to be associated with better outcomes (Baazeem et al. 2011). In our research, four trials evaluated semen parameters including: sperm count, motility and normal morphology. There is no significant difference in improvement of sperm parameters between LESS-V and CTL-V groups in each study. However, in these studies, both LESS-V and CTL-V groups could improve sperm parameters. Another important advantage of LESS is subjectively improved cosmesis, one of the driving forces in the development of LESS surgery (Wang et al. 2013). Pooling the data showed significantly better cosmetic satisfaction in the LESS-V group than the CTL-V group. Another study reported verbal response scale and numeric scale to depict cosmetic results. These two scales all showed significantly greater satisfaction in the LESS-V group than the CTL-V group.

Our systematic review has several limitations. There is great heterogeneity among studies for some parameters. Multiple strategies were applied to identify studies, strict criteria to include and evaluate the methodological quality of the studies, and subgroup analysis to minimize the heterogeneity. Future large well-designed studies are needed to address the effect of LESS-V and its longer-term clinical outcomes.

## Conclusion

Our meta-analysis of three RCTs and three retrospective studies including 460 patients comparing the efficacy of LESS-V and CTL-V showed that LESS-V was safe, with significantly reduced postoperative pain, shorter recovery time, and better cosmetic outcome. We found no significant differences in postoperative complications, hospital stay and improvement of sperm parameters. Large-samples, multi-center, well-designed RCTs with complete follow-up data are required to address and update the findings of this analysis in the future.
